# Research progress toward arthropod salivary protein vaccine development for vector-borne infectious diseases

**DOI:** 10.1371/journal.pntd.0012618

**Published:** 2024-12-05

**Authors:** Yuchen Wang, Lin Ling, Lijie Jiang, Alejandro Marin-Lopez

**Affiliations:** 1 Department of Inspection and Quarantine, Shanghai Customs College, Shanghai, China; 2 Section of Infectious Diseases, Department of Internal Medicine, Yale University School of Medicine, New Haven, Connecticut, United States of America; University of Pittsburgh, UNITED STATES OF AMERICA

## Abstract

Hematophagous arthropods, including mosquitoes, ticks, and flies, are responsible for the transmission of several pathogens to vertebrates on whom they blood feed. The diseases caused by these pathogens, collectively known as vector-borne diseases (VBDs), threaten the health of humans and animals. In general, attempts to develop vaccines for pathogens transmitted by arthropods have met with moderate success, with few vaccine candidates currently developed. Nowadays, there are vaccine candidates under clinical trials, including different platforms, like mRNA, DNA, recombinant viral vector-based, virus-like particles (VLPs), inactivated-virus, live-attenuated virus, peptide and protein-based vaccines, all of them based on the presentation of pathogen antigens to the host immune system. A new approach to prevent VBDs has arose during the last decades, based on the design of vaccines that target vector-derived antigens. The salivary secretions of arthropods, in addition of causing allergic reactions and harbor pathogens, are also involved in the transmission and infection establishment in the host, altering its immune responses. In this review, we summarize the achievements in the arthropod salivary-based vaccine development for different vector-borne infectious diseases. This provides a rationale for creating vaccines against different types of arthropod salivary proteins, such as mosquitoes, ticks, and sand flies. Using salivary proteins of clinically important vectors might contribute to achieve protection against and control multiple arthropod-borne infection diseases.

## Introduction

Hematophagous arthropods play a crucial epidemiological role in the transmission of a wide variety of pathogens to the vertebrates they feed on [1]. More than half of the population of the world is at risk for infection with vector-transmitted diseases. These emerging and re-emerging diseases present a considerable public health concern worldwide [2], since the vector geographic distribution is actively expanding, due to the climate change, urbanization, global transportation, and other human-related activities, leading to the appearance of some of these diseases in new regions during the past decades [3]. To control and monitor vector-borne disease expansion, World Health Organization (WHO) has announced new initiative aim to raise the global alarm on the risk epidemics of arboviruses and potential risk of pandemics [4].

Approximately 80% of the global human population is currently vulnerable to one or more vector-borne diseases (VBDs), including dengue, yellow fever, Japanese encephalitis, chikungunya, malaria, leishmaniasis, human African trypanosomiasis, Chagas disease, and onchocerciasis, among others. All these diseases are caused by parasites, viruses, and protozoa that are transmitted by many types of vectors, such as mosquito, aquatic snails, blackflies, fleas, lice, sandflies, ticks, triatome bugs, and tsetse flies, which threaten the health of humans and animals [5]. Among them, around 20% of the worldwide burden of tropical infectious diseases is transmitted through these vectors, mostly affecting areas with inadequate healthcare resources and substandard sanitary [6].

Arthropod-borne virus (arbovirus) is an ecological term defining viruses that are maintained in nature through biological transmission between a susceptible vertebrate host and a hematophagous arthropod, where mosquitoes, ticks, sand flies are the most common vectors [7]. Most of these viruses cause zoonoses that usually depend on nonhuman animal species for maintenance in nature [8]. In the past 40 years, arboviruses have emerged as a major global health problem with the resurgence of several well-known arboviruses, such as Zika virus (ZIKV) [9], West Nile virus (WNV) [10], dengue virus (DENV) [11], or chikungunya virus (CHIKV) [12]. ZIKV outbreaks occurred primarily in the Yap Islands (2007), French Polynesia (2013 to 2014), and South America (2015 to 2016), followed by the states of Rajasthan and Madhya Pradesh in 2018 [13]. Recently, there has been an increase in WNV in southern Europe, with new cases reported in more northern regions [14]. Dengue’s geographical distribution includes 128 countries worldwide, affecting 390 million people every year causing significant morbidity and mortality in children and adults everywhere [15]. During the 2006 La Réunion outbreak, a large number of travelers from industrialized countries became infected with CHIKV, remaining infected when they returned home, and dispersing the disease to other countries. In 2007, CHIKV was identified in Italy, where a total of 205 cases of confirmed CHIKV infection were reported between 4 July and 27 September 2007 [16]. Other outbreaks were later reported in Malaysia in 2008 to 2009 and Bangladesh in 2008, where the CHIKV isolates were similar to the variants isolated in Cameroon, Indian Ocean islands, India, Italy, and Gabon [17]. Arboviruses can also be transmitted by ticks. Approximately 10,000 to 12,000 clinical cases of tick-borne encephalitis (TBE) are reported each year [18], but this estimation is believed to be significantly lower than the actual total number of clinical cases. Tick borne encephalitis virus (TBEV), Crimean–Congo hemorrhagic fever virus (CCHFV), severe fever with thrombocytopenia syndrome virus (SFTSV), Powassan virus (POWV), or African swine fever virus (ASFV) are some of the representatives of this group, showing a strong zoonotic potential.

Parasites and bacteria can also be transmitted by many types of arthropod vectors. According to the latest Report by World Health Organization, the African Region continues to carry a disproportionately high share of the global malaria burden, the most prevalent arthropod-transmitted disease in the world. A total of 249 million cases of malaria in 2022 were reported by the WHO’s World Malaria Report 2022, resulting in 608,000 deaths in total, mainly affecting children under the age of 5 years. Arthropod-borne bacterial diseases are among the earliest vector-borne illnesses described, like plague, caused by the microbe *Yersinia pestis* and transmitted by *Xenopsylla* fleas [19]. Other highly virulent vector-borne bacterial diseases have been described, such as Rocky Mountain spotted fever and epidemic typhus caused by *Rickettsia rickettsii* (*R*. *rickettsia)* [20] and *Rickettsia prowazekii* (*R*. *prowazekii*) [21], respectively, as well as the Lyme disease [22]. Among arthropod-borne parasites, Plasmodium, the malaria agent, is probably the most spread arthropod borne pathogen in the world, which complete transmission cycle in culicine was initially elucidated by Ronald Ross in 1897 [23]. The protozoan parasite, *Leishmania*, can be transmitted by the bite of infected female sandflies (genera *Phlebotomus* and *Lutzomyia*), with an estimation of 700,000 to 1 million new annual cases [24]. Another medically important parasite is *Trypanosoma brucei* (*T*. *brucei*), known to be the causative agent of the “sleeping sickness” and transmitted by the bite of the *tsetse* fly (*Glossina* species). In the Americas, about 6 to 8 million people are estimated to be infected with *Trypanosoma cruzi* (*T*. *cruzi*), the parasite that causes Chagas disease [25], transmitted by triatomine bugs (genus *Triatoma* and others).

Despite the fact that many important emerging and re-emerging VBDs are becoming better controlled [3], only a few vaccines against VBDs have been licensed for use in humans, like the ones for yellow fever, dengue fever, Japanese encephalitis, and tick-borne encephalitis. However, some are hurdling with safety issues and present limitations based on age, previous pathogen exposure, and immunological status. Notably, different platforms have been evaluated for the design of vaccines [26], including DNA, mRNA, viral vectors, virus-like particles (VLPs), inactivated virus, live attenuated virus, peptide and protein-based vaccines, passive immunizations by using monoclonal antibodies (MAbs), and vaccines that target vector-derived antigens [27]. Conventional pathogen-based vaccines struggle against the high genetic variability of viruses and the intricate life cycles of parasites or bacteria. The lack of effective preventive vaccines and therapeutic measures remains a crucial issue. Consequently, creating dependable vaccines becomes an exceedingly difficult task in the face of such complexity. An approach worth considering involves directing attention to the interface between the vector and host, achieved by integrating vector salivary proteins into vaccines designed to counter pathogens. This is based on the observation that vector saliva and some specific salivary factors facilitate pathogen transmission from the mosquito to the host [28]. However, controversy persists over whether exposure to vector saliva worsens or safeguards against severe clinical symptoms, induces immunity through natural exposure, or applies universally across all vector species and associated pathogens [29]. Despite ongoing debates, harnessing the potential of this distinctive biological aspect stands as a promising and practical strategy for the development of vaccines targeting diseases transmitted by vectors [29].

Since the life cycle of arboviruses is highly dependent on arthropods, control of the arthropods (vectors) is critical for the control of arbovirus infection [30]. It has been widely observed that the salivary secretions of arthropods can cause allergic reactions in host vertebrates or harbor pathogens. Potential biological and epidemiological applications of immunogenic salivary molecules are being explored: they can be used as biomarkers of vector and arbovirus exposure or used as vaccine candidates that are liable to improve host protection against vector-borne diseases [31]. However, further studies are necessary to understand the molecular mechanisms of the immune alterations that the arthropod salivary secretions induce in the host, which is a key step for the vector-based vaccine development [32]. This review covers known vaccine candidates that target the arthropod salivary gland components, with emphasis on the most common 3 arthropod vectors: mosquitoes, ticks, and sandflies.

## The importance of arthropod salivary factors in pathogen transmission

Saliva from hematophagous arthropods contains a variety of bioactive components [33], which are designed to modulate the host immune response to facilitate blood feeding. Alterations in coagulation, hemostasis, cytokine production, or flux of immune cells, among others, can contribute to the pathogenesis of viruses and other microorganisms, leading to enhancement of vector-borne infections. During the blood-feeding process, saliva is introduced into the host, altering homeostasis and triggering a robust immune response [31]. There is evidence that the saliva enhances infection in naïve hosts by hijacking host immune responses, and some studies have shown that prior exposure to saliva results in less severe infection [34]. People who are repeatedly exposed to ticks may develop an immune response to tick salivary proteins, and the presence of anti salivary antibodies can be used as markers of exposure [35]. Several salivary proteins have been shown to be immunogenic, able to induce antibody production, and some of them also exhibit immunomodulatory properties that can enhance arboviral infection [36], and the research have showed the evaluation of antisalivary Ab responses could be a useful approach for identifying a marker for the risk of malaria transmission [37], which means the evaluation of human immune responses to arthropod bites may be a useful biomarker of infection. Based on this, saliva has recently been a focus for vaccine research. Today, many salivary molecules have been identified and characterized as new targets for the development of vaccines against arthropod-borne pathogens [38]. In the pursuit of vaccine development, researchers are exploring the protective effects of long-term exposure to insect vector saliva, aiming to address the challenges posed by these diseases [39].

Many salivary factors have been characterized and their immunological role in infection determined. The saliva of hematophagous arthropods contains a toolbox of anti-hemostatic, anti-inflammatory, and immunomodulatory molecules that contribute to the success of the blood meal. At the molecular level, this is reflected by the existence of various pharmacologically active molecules in arthropod saliva, which are employed to face the constraints of vertebrate host hemostasis, inflammation, and adaptive immunity [31]. Some arthropods exhibit salivary components which inhibit host vasoconstrictor agents, including peroxidase [31]. Other arthropods secrete strong vasodilators in their saliva, to increase blood flow in the capillaries that irrigate the skin [40]. Damage caused by the bite also generates a host response, like the activation of platelets and clot formation [41]. To counteract this, mosquitoes have evolved factors like apyrase, an enzyme that degrades ADP released by activated platelets [42]. Alterations in inflammation driven by saliva components are also observed. Many factors are directed to inhibit the effect of pro-inflammatory mediators, which activate and recruit leucocytes at the bite site [43]. Saliva also leads to both Type 1 and Type 4 immune hypersensitivity reactions, classically associated with allergic diseases [44]. Additionally, many salivary proteins are immunogenic and induce humoral and cellular responses in mammalian hosts [45]. Research indicated that hematophagous arthropod saliva can greatly impair the development of an appropriate adaptive immune response by the host by altering the function of antigen presenting cells (APC), such as macrophages [46] or dendritic cells (DC) [47]. In general, salivary gland extracts (SGEs) from several hematophagous arthropods can suppress the secretion of Th1 cytokines, such as IFN-γ and IL-2, thus facilitating the development of an antibody-mediated Th2 response [31]. This polarization of host immunity toward a Th2 response (to the detriment of a Th1 cell-mediated response) is advantageous for successful blood feeding, but it may also positively affect pathogen transmission [31]. Moreover, it has been reported that mosquito saliva consistently suppressed inducible nitric oxide synthase (iNOS) expression in APCs, especially macrophages [48]. The catalytic activity of iNOS produces the reactive oxygen intermediate NO, which causes smooth muscle relaxation, inhibition of platelet activation, and induces direct and indirect immune responses. The expression of iNOS is also associated with the regulation of a broad variety of transcription factors, including NF-κB, STAT-1α, and IFN regulatory factor-1 (IRF-1) [49].

All these alterations in the local immune environment can lead to an increase in arthropod borne pathogen-susceptible cells at the infected bite site [50]. A salivary antigen, *Anopheles gambiae* (*An*. *gambiae*) sporozoite-associated protein (AgSAP), has been found to directly interact with the *Plasmodium falciparum* and *Plasmodium berghei* sporozoites by binding to heparan sulfate and inhibiting local inflammatory responses in the skin [51]. This binding seems to facilitate early *Plasmodium* infection in the vertebrate host, serving as a target for the prevention of malaria [51]. A vector protein, with similarity to the human gamma interferon inducible thiol reductase (GILT), was found in mosquito saliva and referred as mosquito GILT (mosGILT). This anopheline factor is also associated with sporozoites and inhibits their ability to traverse cells [52]. Numerous studies have demonstrated that mosquito saliva facilitates viral transmission too. *Aedes aegypti* (*Ae*. *aegypti*) salivary factor, AaSG34, has been associated with an increase in DENV2 replication [53]. Another saliva-specific protein, named *Ae*. *aegypti* venom allergen-1 (AaVA-1), promotes DENV and ZIKV transmission by activating autophagy in host immune cells of the monocyte lineage [54]. An *Ae*. *aegypti* 15-kilodalton salivary protein, named LTRIN, was shown to facilitate the transmission of ZIKV and exacerbated its pathogenicity by interfering signaling pathways mediated by the lymphotoxin-β receptor (LTβR) [55]. Another salivary protein from *Ae*. *aegypti* mosquito, named *Ae*. *aegypti* bacteria-responsive protein 1, AgBR1, was found to stimulate splenocyte expression of inflammatory factors like IL-1b, induce the infiltration of neutrophils at the mosquito bite site, and enhance the pathogenic mechanism of ZIKV in the immunocompromised AG129 mouse model [56]. Another mosquito salivary protein, known as neutrophil stimulating factor 1, Nest1, has also been found to enhance ZIKV disease by inducing the expression of cytokines (such as IL-1b, Cxcl2, and Ccl2 chemokines) in mouse neutrophils [50].

Tick saliva also contains proteins with antihemostatic, anti-inflammatory, and immunosuppressive properties. These proteins serve to counteract the host’s hemostatic, immune, and inflammatory responses, creating a conducive environment for the tick to feed on host blood and promoting the successful infection of the host by tick-borne pathogens. An *Ixodes scapularis* (*I*. *scapularis*) tick salivary protein, Salp15, was shown to bind to the CD4 receptor on the surface of T lymphocytes. This interaction interferes with TCR-mediated signal transduction, inhibiting CD4^+^ T cell activation and proliferation. Salp15 also exhibits specific binding to dendritic cells, interacting with the dendritic cell-specific intercellular adhesion molecule-3-grabbing non-integrin (DC-SIGN) protein and up-regulating the expression of CD73 in regulatory T cells [57]. Another *I*. *scapularis* tick salivary protein, IsC1ql3, can interact with the globular C1q receptor present on the surface of CD4^+^ and CD8^+^ T cells, resulting in decreased production of interferon γ. These alterations in the host immune response led to the increase in *Borrelia burgdorferi* (*B*. *burgdorferi*) infectivity observed in the mouse model [58].

Saliva of other hematophagous arthropods like sand flies can also alter immune responses in the host and enhance infection levels of vector-transmitted pathogens like *Leishmania* [59]. Sand fly saliva enhances *Leishmania amazonensi*s infection by increasing IL-10 production in draining lymph nodes. Tsetse fly Saliva has also been shown to trigger the onset of *T*. *brucei* parasitemia in a mouse model, which correlates with a higher mortality rate and a reduced host inflammatory response [60]. Saliva of the Triatominae bug *Rhodnius prolixus*, known vector of Chagas disease, also contributes to *T*. *cruzi* infection. A factor contained in this saliva, lysophosphatidylcholine (LPC), can attenuate proinflammatory cytokine expression, NO production and promotes *T*. *cruzi* association with macrophages [61].

Some salivary proteins elicit a strong antibody response that is related to the intensity of vector bites. This phenomenon set up the base of the study of antibody immune response against salivary factors as a marker of vector exposure, which has been evaluated for many vectors like ticks and mosquitoes. Factors like calreticulin from *Ixodes* ticks or Nterm-34 kDa and D7 proteins in *Aedes* and *Anopheles* mosquitoes have been proposed as markers of exposure [35,36,62,63]. Additionally, other authors showed a correlation between antibody responses against vector saliva and certain salivary factors like AgBR1 and Nterm-34 kDa and risk of infections like dengue and malaria [36,37]. These observations made possible the evaluation of human immune responses to arthropod bites as useful biomarker of infection.

Salivary components can modulate host immunity at the bite site and induce an immune environment favorable for pathogen transmission. This immuno-modulation is associated with the production of specific antibody responses to counteract saliva effects. Therefore, and based on these observations, the generation of vaccine candidates based on arthropod salivary components, summarized in Table 1, could be a strong tool to prevent arthropod-borne infections at different stages, like transmission, dissemination, or infectivity.

**Table 1:** Arthropod salivary protein vaccine candidates for vector-borne infectious diseases.

Type of the vector	Vector species	Salivary molecule	Immunization strategy	Model used for the study	Condition or disease	Bibliographic references (PMID)
Mosquito	*Ae*. *aegypti*	NeSt1	Stimulates neutrophils	In vitro, in vivo (mice)	Zika virus	30971475
	*Ae*. *aegypti*	AgBR1	Suppress early inflammatory responses	In vitro, in vivo (mice)	Zika virus, West Nile virus	30858571
	*Ae*. *aegypti*	LTRIN	Interfere with signaling through the LTβR	In vitro, in vivo (mice)	Zika virus	29507355
	*Cx*. *tarsalis*	SGE	Produce increased levels of Th1-type cytokines (IFNγ and TNFα)	In vitro, in vivo (mice)	West Nile virus	23362833
	*An*. *gambiae*	AGS-v	Prompt a Th1 response	In vitro, in vivo (Clinical trial, Phase I)	Zika virus	36436281
	*Anopheles*	SAMSP1	Decrease neutrophil chemotaxis	In vitro, in vivo (mice)	*Plasmodium* infection	33306099
	*An*. *gambiae*	AgTRIO	Decrease parasitemia	In vitro, in vivo (mice)	*Plasmodium* infection	29649443
Tick	*Ixodes*	Salp15	Interferes with TCR-mediated signaling transduction	In vitro, in vivo (mice)	*B*. *burgdorferi* infection	31998324
	*I*. *scapularis*	Salp25D	Decrease *Borrelia* acquisition by *I*. *scapularis*	In vitro, in vivo (mice)	*B*. *burgdorferi* infection	18005713
	*I*. *scapularis*	TSLPI	Inhibits the human lectin complement pathway	In vitro, in vivo (mice)	*B*. *burgdorferi* infection	21843870
	*I*. *scapularis*	19ISP	Protect mRNA from degradation	In vitro, in vivo (guinea pigs)	*B*. *burgdorferi* infection	34788080
	*I*. *scapularis*	IsC1ql3	Inhibits *B*. *burgdorferi*-induced IFN-γ production	In vitro, in vivo (mice)	*B*. *burgdorferi* infection	36417869
	*I*. *scapularis*	Salp14	Inhibiting the coagulation factor Xa	In vitro, in vivo (guinea pigs)	Tick infection	34862075
	*O*. *moubata*	APY	Anti-haemostatic activity	In vitro, in vivo (rabbits)	Tick infection	26293586
	*O*. *moubata*	MOU	Anti-haemostatic activity	In vitro, in vivo (rabbits)	Tick infection	26293586
	*O*. *moubata*	Om44	Prevent the adhesion of leucocytes and platelets to vessel walls	In vitro, in vivo (pigs)	Tick infection	19735664
	*I*. *ricinus*, *I*. *scapularis*	TSGPs	Associated with tick immunity against both *I*. *scapularis* and *I*. *ricinus*	In vitro, in vivo (rabbits)	TBEV-infection, *B*. *burgdorferi* infection, *Babesia* infection	36357287
	*I*. *scapularis*	EL-6	Activat of arthropod Toll pathway	In vitro, in vivo (mice)	*A*. *phagocytophilum* infection	37253745
Sand fly	*L*. *longipalpis*	LJM19	Produce increased levels of IFN-γ/TGF-β ratio and inducible NOS expression	In vitro, in vivo (hamsters)	*Leishmania* infection	18509051
	*L*. *longipalpis*,	LJM11	Inducing a Th1 immune response	In vitro, in vivo (mice)	*Leishmania* infection	22739793
	*P*. *duboscqi*	PdSP15	Correlate to the early appearance of *Leishmania*-specific CD4(+)IFN-γ(+) lymphocytes	In vitro, in vivo (rhesus macaques)	*Cutaneous leishmania* infection	26041707
	*P*. *papatasi*	PpSP15	Induce a Th1-biased cellular immunity	In vitro, in vivo (mice)	*Leishmania major* infection	32409684

## Salivary protein vaccines for mosquito-borne diseases

Mosquitoes are the major vectors for arthropod borne pathogens. Mosquito-borne diseases constitute a large portion of infectious diseases, causing more than 700,000 deaths annually [64]. Several mosquito salivary factors have been tested as potential vaccine candidates. Passive immunization against the antigenic *Ae*. *aegypti* factor NeSt1, prevents (AG129) mice from early ZIKV replication and increases survival rates in ZIKV-infected *Ae*. *aegypti* mosquitoes [50]. Passive and active immunizations against another *Ae*. *aegypti* salivary factor, AgBR1 also partially protected mice from lethal mosquito-borne-ZIKV infection [56]. Passive immunization against this antigen also reduced WNV infection in a WNV-*Ae*. *aegypti* laboratory infection model [65]. Additionally, combination with specific sera against these 2 proteins was also tested, enhancing survival, and reducing viremia [66]. Passive transfer of antibodies against LTRIN salivary protein also protected mice against ZIKV infection, reducing ZIKV burden in target tissues like the brain [55]. Another study showed that mice immunized with *Culex tarsalis* (*Cx*. *Tarsalis*) SGEs produced increased levels of Th1-type cytokines (IFNγ and TNFα) after challenge with mosquito-transmitted WNV and exhibited both a delay in infection of the central nervous system (CNS) and significantly lower WNV brain titers compared to control mice [67]. These studies suggest that development of a mosquito salivary-based protein vaccine candidates might be a strategy to consider in order to control arthropod-borne viral pathogens such as ZIKV, DENV, and WNV.

*Anopheles* mosquito salivary components have also tested as vaccine candidates against malaria. *An*. *gambiae* saliva vaccine (AGS-v) is a synthetic vaccine composed of 4 salivary peptides derived from *An*. *gambiae* salivary gland factors. These are also common in other vector species like *Aedes* spp. and *Culex* spp. In a first-in-human Phase 1 trial, AGS-v showed to be immunogenic. An extended study adding a fifth peptide to the AGS-v composition, AGS-v PLUS, can also prevent ZIKV infection in vitro [68]. Other candidates are also under study, like the sporozoite-associated mosquito saliva protein 1 (SAMSP1), a mosquito saliva protein that can influence sporozoite infectivity in the vertebrate host. Active and passive immunization with SAMSP1 or SAMSP1 antiserum diminished the initial *Plasmodium* burden after infection in the mouse model [69]. Antibodies to *An*. *gambiae* TRIO (AgTRIO) contributes to protection against *Plasmodium* infection in mice [70]. In addition, active immunization studies using AgTRIO mRNA-lipid nanoparticles also induced protection, stimulating the generation of IgG2a isotype antibodies [71].

## Salivary protein vaccines for tick-borne diseases

Ticks are obligate hematophagous ectoparasites of wild and domestic animals as well as humans, considered the world’s second largest vector of human disease after mosquitoes [72]. Increases in tick distribution and tick-borne disease prevalence and transmission, mainly due to climate change are important public health issues [73]. This increase hinders the efforts made to control the expansion of emerging diseases transmitted by ticks [74]. Tick-borne diseases are an important cause of human morbidity and mortality, including Lyme disease, tick-borne relapsing fever (TBRF), anaplasmosis, ehrlichiosis, spotted fever rickettsioses, babesiosis, TBE, and viral hemorrhagic and encephalopathy fevers [75].

Intensive basic research in the field of tick salivary gland transcriptomics and proteomics has identified several major protein families that play important roles in tick feeding and overcoming vertebrate anti-tick responses [76]. Anti-tick host immune responses are heightened upon repeated tick exposure and have the potential to abrogate tick salivary protein function, interfere with the blood meal and prevent pathogen transmission [77]. Progress in the development of anti-tick vaccines for humans has been slow due to the complexities of such vaccines but has recently accelerated with some potential candidates being under study.

Passive transfer studies with specific antiserum against the CD4^+^T cell inhibitor Salp15 were shown to significantly protect mice from *B*. *burgdorferi* infection [78,79]. This antibody-based therapy against Salp15 was also tested in combination with specific antibodies against *B*. *burgdorferi* antigens, such as OspA or OspC. This protective effect could be a consequence of phagocytosis enhancement, as in vitro assays showed that Salp15 antiserum increased the clearance of Salp15-coated *B*. *burgdorferi* by phagocytes, suggesting a potential mechanism of action [79]. Another member of the Salp group, Salp14, an anticoagulant that inhibits factor Xa, is present in tick saliva and is associated with partial tick immunity. Vaccination with Salp14 mRNA-LNPs elicited erythema at the tick bite site after tick challenge in a guinea pig animal model [80]. Erythema is one of the most important early visible hallmarks of acquired tick resistance and has the potential to generate itching and pain [81]. This clinical sign may facilitate an early recognition and self-removal of the biting tick, may be sufficient to prevent *Borrelia* transmission. [80]. Immunization with another *I*. *scapularis* salivary gland agent, Salp25D, was shown to reduce spirochete acquisition by ticks [82]. Mice vaccinated with the tick salivary lectin pathway inhibitor (TSLPI) was showed to be also protective against *Borrelia* burden, although it did not completely block transmission [83]. Another recent study using mRNA-LNPs as a vaccine platform consisted in the immunization of guinea pigs with 19 different *I*. *scapularis* salivary proteins (19ISP) shown to be immunogenic and have a role during blood feeding. After immunization and tick challenge, this candidate reduced the tick attachment times on the host, important parameter to prevent *B*. *burgdorferi* infection [84]. A probable underlying mechanism could be the induction of a local redness early after *I*. *scapularis* attachment, preventing the ticks to take a normal blood meal [84]. The *Ixodes* factor IsC1ql3, which inhibits *B*. *burgdorferi*-induced IFN-γ production has also been a target for tick salivary based vaccine development. IsC1ql3-immunized mice fed upon by *B*. *burgdorferi*-infected ticks have a lower spirochete burden during the early phase of infection [58]. A similar approach was followed targeting other salivary proteins in the eyeless tampan tick (*Ornithodoros moubata*, *(O*. *moubata)*), main vector of ASFV and the TBRF [85]. Phospholipase A2 (PLA2), a 7DB-like protein (7DB-like), a riboprotein 60S L10 (RP-60S), an apyrase (APY), and a new platelet aggregation inhibitor peptide, designated mougrin (MOU) were used as vaccine candidates in rabbits [86]. Tick saliva inoculated during natural tick-host contacts had a boosting effect on vaccinated animals, increasing specific antibody levels and protection [86]. Another salivary antigen described to induce anti-tick immune responses was Om44, which binds to host P-selectin in the host. Immunization based on this antigen inhibited feeding and produced alterations in other tick activities like fecundity [87]. A different approach to combat tick infestation was based on the target of gut antigens, like the Bm86 from *Rhipicephalus microplus* (*R*. *microplus*, formerly *Boophilus microplus*) which is commercially available as an anti-tick vaccine (Gavac), which was 55% to 100% effective in controlling *Boophilus microplus* (*B*. *microplus*) infestations in grazing cattle 12 to 36 weeks after the first vaccination, and it is widely used to prevent cattle against multiple tick infestations in numerous countries [88].

Tick salivary antigens have been also used as vaccine candidates against TBEV, endemic in several European countries, showing and increasing incidence [89]. It has been reported that the human TBEV infection can be prevented by vaccines targeting the virus directly [90], but require multiple doses and frequent boosters to induce and maintain immunity [91]. Therefore, the use of tick salivary targets has emerged as an attractive additional or alternative prophylactic measure. Immunization based on the 15-KDa protein named 64TPR, which is a tick cement protein expressed at higher levels during tick feeding, showed protection in mice from TBEV, with a reduction of 50% in the viral titers [92]. Moreover, 64TPR has been shown as a broad-spectrum vaccine candidate against several tick species, including *Rhipicephalus sanguineus* (*R*. *sanguineus*), *Ixodes ricinus* (*I*. *ricinus*), *Amblyomma variegatum* (*A*. *variegatum*), and *B*. *microplus* [93]. Tick immunity is mainly directed against tick salivary gland proteins (TSGPs) and has been shown to partially protect against experimental Lyme borreliosis [94]. It has been identified that one of the TSGPs protected against Lyme borreliosis by actively immunize mice and 2 TSGPs that, when tested as antigens for an anti-tick vaccine, partially protected mice from lethal TBEV infection according to an ANTIDotE project (Grant Identification Code: 602272). There is currently no vaccine to prevent Lyme borreliosis, but a refined vaccine containing protective epitopes from *Borrelia* species outer surface protein A (OspA) serotypes in healthy adults aged 18 to 70 years has recently been tested in Phase I/II trials, which are registered with ClinicalTrials.gov, number NCT01504347. The novel multivalent OspA vaccine could be an effective intervention for the prevention of Lyme borreliosis in Europe and the United States, and perhaps worldwide [95].

Ticks are also able to transmit parasites like *Anaplasma phagocytophilum* (*A*. *phagocytophilum*), aetiologic agent of human anaplasmosis [96], which is transmitted by *I*. *scapularis* [97]. Passive immunization using EL-6 antibody (raised against the C-terminal Extracellular Loop-6 of IsOATP4056 protein) impaired the transmission of *A*. *phagocytophilum* from ticks to the murine host and also reduced bacterial loads in engorged ticks [98].

## Salivary protein vaccines for sand fly-borne diseases

Sand flies are another group of hematophagous insects responsible for the transmission of vector-borne diseases to humans and animals. The most prominent of these diseases is Leishmaniasis, which affects the skin and mucous surfaces as well as organs such as the liver and spleen [99]. Phlebotomine sand flies are known to transmit leishmaniasis, but also bacteria and viruses like members of the *Phlebovirus*, *Vesiculovirus*, and the *Orbivirus* families [100].

Transcriptomic and proteomic studies have enumerated the repertoire of sand fly salivary proteins that can be used either as biomarkers of vector exposure or as anti-Leishmania vaccines [101,102]. Furthermore, they have been useful to cluster protein families that are unique or conserved, useful for the generation of taxon-specific biomarkers of vector exposure, or pan-specific vaccines, respectively [103]. Immunity to sand fly salivary proteins has been shown to protect rodents and nonhuman primates against Leishmania infection [104,105]. Combinatorial strategies utilizing *Leishmania* parasite and salivary factors have been also tested, trying to target the most vulnerable parasite stage just after transmission [106].

Immunization with 16 different DNA plasmids encoding several salivary proteins of *Lutzomyia longipalpis* (*L*. *longipalpis*) resulted in the identification of LJM19, a novel 11-kDa protein, which protected hamsters against the fatal outcome of visceral leishmaniasis, reinforcing the concept of using components of arthropod saliva in vaccine strategies against vector-borne diseases [104]. Another factor from the vector *L*. *longipalpis*, LJM11, has also been tested as a protective vaccine candidate, inducing a Th1 immune response and protect mice against bites of *Leishmania* major-infected sandflies, even conferring long-lasting immunity and ulcer-free protection [107,108].

Salivary products of other sand fly vectors like *Phlebotomus duboscqi* (*P*. *duboscqi*) have been also studied as vaccine candidates. Uninfected sand fly bites or immunization with salivary protein PdSP15 showed protection against cutaneous leishmaniasis in nonhuman primates, may be mediated or related to an early stimulation of *Leishmania*-specific CD4^+^IFN-γ^+^ lymphocytes [105]. Homologs of PdSP15 like PpSP15 have also been found in other *Phlebotomus* species like *P*. *papatasi*, which has been also shown to be protective against *Leishmania* major infection [109].

## Discussion

Mosquitoes, ticks, and sand flies comprise many hematophagous arthropods considered vectors of human infectious diseases. While consuming blood to obtain the nutrients necessary to carry on life functions, these insects can transmit pathogenic microorganisms to the vertebrate host. These include, for example, bacterial diseases like the Lyme agent, arboviruses, and malaria, threatening the health of humans and animals. No effective vaccines are available for many of them, so new approaches to prevent disease produced by these pathogens are being considered. Based on the evidence that arthropod-borne pathogens exploit the immunomodulatory properties caused by vector saliva in the vertebrate host during the transmission process, the design of vaccines based on salivary components could be a good alternative or a complement in combination with classic vaccines for the prevention of arthropod-borne pathogens.

Some of these arthropod factors with demonstrated pro-pathogenic effect have been tested as vaccine candidates in animal models, showing that active and passive immunizations are effective in controlling pathogen replication and reducing clinical signs. This approach offers some advantages compared with traditional pathogen antigen-based vaccines: they are designed to block or reduce pathogen dissemination at early stages of infection, increasing the probabilities of efficacy. Salivary-based vaccine candidates do not place evolutionary pressure on the pathogens during infection, reducing the likelihood of resistance. Arthropod bites in immunized animals can induce a boosted response providing a natural re-immunization to the anti-salivary factor, maintaining protective IgG levels, and limiting the need for booster injections. These types of vaccines can be efficacious against multiple mosquito-borne diseases that share similar life cycles. Anti-salivary antibodies may synergize with anti-virus antigen-antibodies to prevent virus dissemination in the host, showing a potential use of these salivary-based vaccines in conjunction with the currently marketed vaccines. However, only few candidates have been tested for clinical trials or have reached the market. Among them, Gavac and tickGARD, protein vaccines based on the Bm 86 antigen from the cattle tick (*B*. *microplus*) were used to prevent tick infestation in livestock, then also preventing babesiosis cases [110,111]. In addition, Phase 1 clinical studies have been performed to test human safety and immunogenicity of AGS-v and AGS-v PLUS, a vaccine cocktail of Anopheles salivary peptides. This candidate showed to be immunogenic, conferred protection in a malaria mouse model and surprisingly showed anti ZIKV properties in an in vitro system, using peripheral blood mononuclear cells from immunized participants [68,112]. This last observation will require further investigation, since the vaccine composition does not present any substance related to *Aedes* mosquitoes but may contain conserved epitopes or induce immune responses that are also protective against viral infections. There are still multiple questions to resolve and obstacles to overcome in the design of salivary-based vaccines. For instance, most of the molecular mechanisms that govern the salivary antigen–host interactions remain unclarified. Adverse events, especially those related to allergic-type reactive responses against saliva proteins should be also considered. Repeated vector exposure in vaccine response has also been poorly studied, but there are some evidence that demonstrate the booster effect of the mosquito bite [71]. The need of multiple immunizations, especially in areas where the specific mosquito species are not endemic can also limit their efficacy. However, this can be improved with the use of more efficient adjuvants or immunization strategies to promote and maintain a high degree of immune response.

This review brings a summary of the vaccine candidates based on vector components that have been tested against multiple pathogens and different vectors (Table 1). Although there are still drawbacks to overcome, the target of arthropod salivary factors for vaccine development opens a big field for the control of arthropod-borne pathogens, with some human clinical trials ongoing. In addition, these salivary-based candidates could also be complemented with classic pathogen-derived vaccines to increase their efficacy and potentially amplify their spectrum against multiple vector-transmitted microorganisms.
